# Glossopharyngeal Dystonia Secondary to a Lurasidone-Fluoxetine CYP-3A4 Interaction

**DOI:** 10.1155/2013/136194

**Published:** 2013-05-16

**Authors:** Sean Paul, Brian K. Cooke, Mathew Nguyen

**Affiliations:** University of Florida, Gainesville, FL 32606, USA

## Abstract

Acute dystonic reactions are becoming much less prevalent in clinical practice due to the use of newer antipsychotics. Drug-drug interactions, patient characteristics, and environmental and genetic factors all contribute to the rate of occurrence of acute dystonia with second generation agents. In this case, we report a glossopharyngeal dystonia secondary to a lurasidone-fluoxetine CYP-3A4 interaction to highlight the importance of maintaining an index of suspicion for laryngeal dystonia, a potentially fatal dystonia.

## 1. Introduction

Both first-generation antipsychotics (FGA) and second-generation antipsychotics (SGA) have equal efficacy in treating positive symptoms of schizophrenia; however, SGAs are now considered to be first-line agents, mainly because they have less propensity for side effects, including extrapyramidal symptoms (EPS), tardive dyskinesia (TD), weight gain, and metabolic side effects [1, 2]. The rates for EPS for haloperidol, clozapine, and chlorpromazine are estimated at 20%, 12%, and 25%, respectively [2, 3]. However despite choosing a SGA, acute EPS may still occur, with the rates ranging from 1 to 10% depending on the agent [4].

FGAs can cause EPS at a higher rate than SGA [5]. The prototypical high potency and arguable “gold standard” FGA is haloperidol, which is noted to have a prevalence rate of 35–52% for akathisia and 55% for parkinsonism [4]. A 2006 Cochrane meta-analysis of 21 studies including about 1500 individuals with schizophrenia reported a number needed to harm (NNH) of 5 with regard to acute dystonia. An NNH of 5 indicates that of 5 people treated with haloperidol, 1 individual will have an acute dystonic event [3]. Typically, acute dystonia occurs within hours of administration and consists of acute contracture of a muscle group resulting in a very uncomfortable position and significant psychological distress. Although all muscle groups can be involved, the most common are the eyes (oculogyric crisis), the neck (torticollis), tongue (glossopharyngeal) the back, the arms, and the large muscle groups of the legs. Risk factors for acute dystonia include male sex, age less than 35, high potency typical antipsychotics, intramuscular route of delivery, previous dystonic reactions, and recent cocaine use [4].

Over the past 6 years, multiple antipsychotics have been approved by the Food and Drug Administration (FDA) for the treatment of schizophrenia, including paliperidone (2006), iloperidone (2009), asenapine (2009), and lurasidone (2010). Although the incidence of acute dystonia is established to be lower than high-potency FGA, lurasidone causes acute dystonia at a rate of 4.2% [6]. Subtypes of dystonia include glossopharyngeal dystonia and laryngeal dystonia, which have similar clinical presentations; however, the latter can be fatal. In this case report, we describe a patient who had an acute glossopharyngeal dystonia reaction after the first dose of lurasidone. We also discuss the similarity of presentation between the two and treatment implications. 

## 2. Case Report

Mr. F is a 27-year-old Caucasian male with a history of depression and schizophrenia with prominent negative symptoms. Before presenting to our outpatient clinic, Mr. F had been treated with multiple atypical antipsychotics including aripiprazole, olanzapine, quetiapine, and ziprasidone. An adequate trial was given for each agent, but the hallucinations and paranoia continued to be socially impairing. Aripiprazole was discontinued due to a poor response at a dose of 45 mg; olanzapine and quetiapine were discontinued secondary to excessive weight gain, and the patient had an acute glossopharyngeal dystonic event after taking the first few doses of ziprasidone about one year before presenting to our clinic. At that time, an acute allergic reaction was ruled out as the patient maintained blood pressure, lacked any skin involvement, and had no other laboratory abnormalities indicating systemic involvement. The symptoms of reduced air movement and tongue swelling were rapidly reversed with 50 mg of intravenous (IV) diphenhydramine.

Mr. F came to our clinic approximately one year after the initial dystonic event and had not taken medications since that time due to the adverse reaction. At this visit, he had notable social isolation, paranoid ideation, and auditory hallucinations. Due to his psychotic symptoms, it was difficult for Mr. F to perform some of his activities of daily living, including using public transportation and going to the grocery store. He was also requesting treatment for his depression symptoms. Fluoxetine was started and titrated over the next few months to 40 mg. Mr. F's depression partially responded to fluoxetine, but the auditory hallucinations continued to be problematic. Due to the history of weight gain from other antipsychotics, lurasidone was chosen to target his psychotic symptoms. After the first dose of lurasidone 40 mg, Mr. F reported difficulty in swallowing and speaking. En route to the emergency room, Mr. F was given IV diphenhydramine, and the reaction dissipated within minutes. An acute allergy was again ruled out due to lack of systemic involvement, lack of a rash, stable vital signs, and normal laboratory values. Mr. F was discharged home a few hours later with a diagnosis of glossopharyngeal dystonia.

## 3. Discussion

This is a case of a 27-year-old Caucasian male with schizophrenia who had previously developed dystonia while taking an atypical antipsychotic, ziprasidone, who later developed a similar reaction after receiving the first dose of lurasidone. In both cases, the reaction was quickly reversed with IV diphenhydramine with exclusion of any allergy. With the second event, the patient was taking fluoxetine 40 mg when he began lurasidone 40 mg. It is important to note that fluoxetine inhibits CYP-3A4 (Table 1), which is the primary route of metabolism of lurasidone. The fluoxetine may have inhibited the lurasidone metabolism, leading to a higher initial dose of lurasidone than intended. The drug manufacturer recommends decreasing the starting dose of lurasidone to 20 mg when coadministered with a potent 3A4 inhibitor [7]. In addition to this drug-drug interaction, this patient has had a history of sensitivity to SGAs.

Lurasidone, similar to other recently approved antipsychotics like iloperidone and asenapine, is associated with less weight gain and less affect on total cholesterol, prolactin, and glucose than other atypical antipsychotics. Unlike iloperidone and ziprasidone, there is no known increase in the QTc interval with lurasidone or asenapine. Other advantages include a simple daily dosing and a simple titration. Disadvantages include dose-related EPS and sedation. Head-to-head efficacy between lurasidone and other SGAs is unclear. Cost is high in comparison to generic antipsychotics [8].

Laryngeal dystonia is relatively rare, and the incidence is not well characterized. It commonly occurs in conjunction with torticollis, retrocollis, tongue and jaw stiffness, oculogyric crisis, and opisthotonus. Symptoms of laryngeal dystonia include slurred speech, stridor, dyspnea, and subjective distress like clutching the throat. The diagnosis is made with a history of medication exposure or recent increase in dosage and ruling out an allergic reaction or airway obstruction. Symptoms that are highly consistent with laryngeal dystonia are desaturation and inability to perform a glottic challenge like sniffing or a forceful cough [9]. Reported cases of laryngeal dystonia have all been associated with high-dose haloperidol (60–100 mg daily). Laryngeal dystonia is a theorized contributing factor to “phenothiazine death,” which has been a term used to explain deaths not related to any definable cause like cardiac arrhythmia or respiratory problems. There have been only a few documented cases with SGAs [10, 11].

In our case, the patient developed shortness of breath and trouble with speaking. In the absence of stridor, hypoxia, or any other signs of compromise of the airway, the more likely diagnosis is a glossopharyngeal dystonia. Glossopharyngeal dystonia commonly presents with dysarthria due to lingual involvement, dysphagia and can occasionally result in difficult breathing as well as cyanosis. However, due to the overlap of symptoms, it is important to maintain an index of suspicion for laryngeal dystonia and thus treat any acute dystonia rapidly [12]. 

This case demonstrates the potential for drug-drug interactions that could have increased the risk of acute dystonia with the use of lurasidone in a patient that had previous EPS sensitivity from another SGA, reminding clinicians to be mindful of potential drug-drug interactions. A prior history of dystonia on a SGA was the most significant risk factor for this patient to develop a subsequent dystonic reaction. Prior acute dystonia on a SGA should trigger careful review of potential drug-drug interactions and a cautious titration of mediation. Further caution should be given to differentiating between laryngeal dystonia and glossopharyngeal dystonia, as the former has a greater propensity for mortality.

## Figures and Tables

**Table 1:** CYP 3A4 substrates, inhibitors, and inducers.

CYP enzyme	Substrates	Inhibitors^a^	Inducers
3A4	Alprazolam, amiodarone, amitriptyline, ^b^astemizole, bupropion, caffeine, ^b^carbamazepine, cisapride, clarithromycin, clonazepam, codeine, ^b^cortisol, cyclosporin, dapsone, ^b^desmethyldiazepam, ^b^diazepam, ^b^diltiazem, erythromycin, estradiol, ethinylestradiol, fluoxetine, haloperidol, ^b^imipramine, ^b^lidocaine, loratadine, lovastatin, midazolam, nefazodone, nicardipine, nifedipine, omeprazole, ^b^ondansetron, orphenadrine, progesterone, quinidine, rifampin, sertraline, tamoxifen, terfenadine, testosterone, trazodone, triazolam, venlafaxine, ^b^verapamil, and ^b^zolpidem	Cimetidine, erythromycin, fluoxetine, fluvoxamine, indinavir, ketoconazole, naringenin, nefazodone, ritonavir, saquinavir, and sertraline (weak)	Barbiturates, carbamazepine, dexamethasone, phenytoin, rifampin, and St. John's wort

^a^Inhibitory potency varies greatly. ^b^More than one CYP enzyme is known to be involved in the metabolism of these drugs.^5^

Reprinted with permission from the American Psychiatric Publishing Textbook of Psychopharmacology (Copyright 2009), American Psychiatric Association.^6^
